# Mitochondrial genomes of the human pathogens *Coccidioides immitis* and *Coccidioides posadasii*

**DOI:** 10.1093/g3journal/jkab132

**Published:** 2021-04-19

**Authors:** Marcus de Melo Teixeira, B Franz Lang, Daniel R Matute, Jason E Stajich, Bridget M Barker

**Affiliations:** Pathogen and Microbiome Institute, Northern Arizona University, Flagstaff, AZ 86011, USA; Faculty of Medicine, University of Brasília-DF, Brasília, Federal District 70910-3300, Brazil; Robert Cedergren Centre for Bioinformatics and Génomiques, Département de Biochimie, Université de Montréal, Montréal, Quebec H3C 3J7, Canada; Biology Department, University of North Carolina, Chapel Hill, NC 27599, USA; Institute for Integrative Genome Biology, University of California, Riverside, CA 92521, USA; Department of Microbiology and Plant Pathology, University of California, Riverside, CA 92521, USA; Pathogen and Microbiome Institute, Northern Arizona University, Flagstaff, AZ 86011, USA

**Keywords:** Valley fever, Coccidioides, Onygenales, coccidioidomycosis, mitochondrial genomes, phylogenetics, fungal pathogen, group I and II introns

## Abstract

Fungal mitochondrial genomes encode genes involved in crucial cellular processes, such as oxidative phosphorylation and mitochondrial translation, and the molecule has been used as a molecular marker for population genetics studies. *Coccidioides immitis* and *C. posadasii* are endemic fungal pathogens that cause coccidioidomycosis in arid regions across both American continents. To date, approximately 150 *Coccidioides* isolates have been sequenced to infer patterns of variation in nuclear genomes. However, less attention has been given to the mitochondrial genomes of *Coccidioides*. In this report, we describe the assembly and annotation of mitochondrial reference genomes for two representative strains of *C. posadasii* and *C. immitis*, as well as assess population variation among 77 selected genomes. The sizes of the circular-mapping molecules are 68.2 Kb in *C. immitis* and 75.1 Kb in *C. posadasii*. We identify 14 mitochondrial protein-coding genes common to most fungal mitochondria, which are largely syntenic across different populations and species of *Coccidioides*. Both *Coccidioides* species are characterized by a large number of group I and II introns, harboring twice the number of elements as compared to closely related Onygenales. The introns contain complete or truncated ORFs with high similarity to homing endonucleases of the LAGLIDADG and GIY-YIG families. Phylogenetic comparisons of mitochondrial and nuclear genomes show extensive phylogenetic discordance suggesting that the evolution of the two types of genetic material is not identical*.* This work represents the first assessment of mitochondrial genomes among isolates of both species of *Coccidioides*, and provides a foundation for future functional work.

## Introduction

Fungal mitochondrial genomes exist as either linear or circular-mapping molecules and range in size from ∼17.6 kb (*e.g.*, *Schizosaccharomyces pombe* Genbank ID MK618090.1) to well over 200 kb [*e.g.*, 272,238 bp in *Morchella importuna* (Liu *et al.* 2020)]. Fungal mitochondrial genomes usually encode proteins involved in oxidative phosphorylation—the main source of ATP production of the cell—as well as two ribosomal RNA subunits, and a set of tRNAs involved in mitochondrial ribosome translation. More specifically, most fungal mitochondrial protein-coding genes fall into the following classes: seven subunits of ubiquinone oxidoreductase [*nad;* six for Saccharomycotina and fission yeasts (Bullerwell *et al.* 2003)], cytochrome b (*cob*), three subunits of cytochrome oxidase (*cox*) and up to three ATP synthase subunits (*atp*; the presence of *atp8* and *atp9* varies among fungal taxa) (Aguileta *et al.* 2014). A gene encoding a ribosomal protein subunit (*rps3*) is present in most fungal mitochondrial genomes (Korovesi *et al.* 2018). Fungal mitochondrial protein-coding genes may contain additional genes encoding structural RNAs such as ribosomal RNAs (small and large subunit rRNAs *rns* and *rnl*), the RNA subunit of RNase P (*rnpB*), and variable numbers of tRNAs, and are more variable across fungal lineages (Aguileta *et al.* 2014). Notably, the *nad* genes do not have this organizational structure, and tend to be arranged in operon-like structures, with some of the genes overlapping without discernable intergenic regions (*e.g.*, *nad4L* situated upstream of *nad5*, overlapping by one to a dozen or more nucleotides) (Aguileta *et al.* 2014).

Mitochondrial genes in fungi contain highly variable numbers and arrangements of group I and II introns that are inserted in protein-coding as well as rRNA genes (Pogoda *et al.* 2019). For example, *Endoconidiophora* species contain more than 80 mitochondrial introns (Zubaer *et al.* 2018), which can create gene annotation challenges especially when transcriptome data are not available. Both intron groups may contain complete or truncated ORFs that encode either homing endonucleases of the LAGLIDADG and GIY-YIG families, or reverse transcriptases/maturases (Lang *et al.* 2007). If present, these proteins can direct intron transfers within mitochondrial genomes of genetically compatible fungal species, or less frequently across genera and kingdom boundaries (Stoddard 2011). Mitochondrial DNA (mtDNA)-encoded genes are particularly prone to crossing species boundaries via introgression even when nuclear gene flow is restricted (Sloan *et al.* 2017). Intron transfer *via* homing endonucleases may involve genetic co-conversion of flanking exon sequences. Therefore, phylogenetic inference using mtDNA for genes with many introns [*e.g.*, *cox1*, *cob* and *rnl* (Bovers *et al.* 2009; Aguileta *et al.* 2014)] may reflect replacement of coding regions from ongoing intron invasion.

In this study, we focus on describing and annotating the mitogenomes of *Coccidioides immitis* and *C. posadasii* (Ascomycota, Onygenales), which are fungal species endemic to both American continents, and the causative agents of coccidioidomycosis (Barker *et al.* 2019). This disease is most frequently reported in the “Lower Sonoran Life Zone” (LSLZ) in California, Arizona, Texas, and northwestern Mexico (Fisher *et al.* 2007; Benedict *et al.* 2019). However, the organism is found throughout arid and semi-arid areas beyond the limits of the LSLZ (Kollath *et al.* 2019). Both species have complex biogeographic distribution patterns (Fisher *et al.* 2001; Teixeira *et al.* 2019a). *Coccidioides immitis* has been found in California and Baja Mexico as well in eastern Washington state, and each region harbors unique genotypes (Engelthaler *et al.* 2016; McCotter *et al.* 2019; Teixeira *et al*. 2019b). *Coccidioides posadasii* is present throughout the southwestern United States in Arizona, Utah, New Mexico, Texas; and in less well-described regions in Central and South America. The species is structured by an Arizona population, a Texas/Mexico/South America (TX/MX/SA) population, and a Caribbean population (Teixeira *et al.* 2019a).

Notably, nucDNA studies have found extensive differentiation between species of *Coccidioides* with some evidence for gene flow between species (Neafsey *et al.* 2010; Maxwell *et al.* 2019). However, no studies have addressed whether or not mtDNA reflects the divergence of nucDNA, or if mtDNA has moved between *Coccidioides* species or among populations. The only precedent suggests that the two species can be discriminated based on polymorphisms found at the first intron of the *cox1* gene (Hamm *et al.* 2019). Therefore, to address these gaps in our knowledge we: (i) describe the full circular mitogenomes of *C. posadasii* and *C. immitis*, (ii) compare their core genes, structural RNAs, and introns of group I and II with other Onygenales fungal species, and (iii) compare the evolutionary histories of the mtDNA and nucDNA genomes of these medically important fungal pathogens.

## Materials and methods

### Mitochondrial genome assembly and annotation

Paired end Illumina sequence reads from 20 *Coccidioides immitis* and 57 *C. posadasii* were retrieved from the Sequence Read Archive (SRA) and accessions and details are listed in Table 1. Following cleaning and quality-clipping of reads with Trimmomatic v0.35, we assembled the genomes of *C. posadasii* Tucson-2 and *C. immitis* WA221 using the SPAdes Genome Assembler v3.14.0 (Bankevich *et al.* 2012) with a kmer sizes 61, 91, and 127. We identified mitochondrial contigs in this initial assembly using similarity searches with expected mtDNA-encoded fungal proteins (*cob, cox1, nad1, nad2, nad3, nad4, nad4L, nad5, nad6, atp6*, and *atp9* of *Allomyces macrogynus*; NCBI Reference Sequence NC_001715.1). To minimize assembly error we (i) used Rcorrector (Song and Florea 2015) read correction, (ii) reduced the number of Illumina reads to a target kmer coverage of the mtDNA between 30 and 50x, (iii) reads mapping against the identified mitochondrial contigs were identified with Bowtie2 (Langmead and Salzberg 2012), which were then (iv) reassembled with Spades, resulting in preliminary (uncorrected) mitogenome assemblies. Finally, (v) all reads of the reduced 30–50x read set were aligned back to the preliminary assembly with Bowtie2 and analyzed for kmer coverage with Bedtools v2.29.2 (Quinlan 2014). We identified incorrectly assembled reads, defined by kmer frequency values of two or lower (likely the result of hybrid reads, originating from ligation of unrelated genomic DNA fragments during library construction), and removed them from the final assemblies. The circularity of the final mitogenomes assemblies was verified by identifying overlapping regions present on both ends of the final contigs. For both species, we obtained single circular-mapping closed contigs that carry the expected full set of fungal mitochondrial genes.

**Table 1:** SRA accession number, isolate ID, species, and place of isolation for each strain used for nuclear and mitochondrial genetic analyses

SRA accession number	Isolate ID	Species	Country of isolation
SRR6830881	4545-MICE	*Coccidioides posadasii*	Venezuela
SRR6830884	4542	*Coccidioides posadasii*	Venezuela
SRR6830886	3796	*Coccidioides posadasii*	Venezuela
SRR6830885	2566	*Coccidioides posadasii*	Venezuela
SRR6830888	JTORRES	*Coccidioides posadasii*	Venezuela
SRR6830887	3490	*Coccidioides posadasii*	Mexico
SRR3468076	730332_Guatemala	*Coccidioides posadasii*	Guatemala
SRR3468077	730333_Guatelama	*Coccidioides posadasii*	Guatemala
SRR3468078	730334_Guatemala	*Coccidioides posadasii*	Guatemala
SRR3468067	B0858_Guatemala	*Coccidioides posadasii*	Guatemala
SRR3468068	B10757_Nevada	*Coccidioides posadasii*	United States
SRR3468069	B10813_Texas	*Coccidioides posadasii*	United States
SRR3468070	B1249_Guatemala	*Coccidioides posadasii*	Guatemala
SRR3468072	B5773_Brazil	*Coccidioides posadasii*	Brazil
SRR3468053	Coahuila_2	*Coccidioides posadasii*	Mexico
SRR3468050	Colorado_Springs_1	*Coccidioides posadasii*	United States
SRR3468075	GT002_Texas	*Coccidioides posadasii*	United States
SRR3468074	GT017_Paraguay	*Coccidioides posadasii*	Paraguay
SRR3468066	Michoacan_1	*Coccidioides posadasii*	Mexico
SRR3468064	Nuevo_Leon_1	*Coccidioides posadasii*	Mexico
SRR3468065	Nuevo_Leon_2	*Coccidioides posadasii*	Mexico
SRR3468054	Phoenix_1	*Coccidioides posadasii*	United States
SRR3468055	Phoenix_2	*Coccidioides posadasii*	United States
SRR3468056	Phoenix_3	*Coccidioides posadasii*	United States
SRR3468057	Phoenix_4	*Coccidioides posadasii*	United States
SRR3468058	Phoenix_5	*Coccidioides posadasii*	United States
SRR3468059	Phoenix_6	*Coccidioides posadasii*	United States
SRR3468061	Phoenix_7	*Coccidioides posadasii*	United States
SRR3468062	Phoenix_8	*Coccidioides posadasii*	United States
SRR3468063	Phoenix_9	*Coccidioides posadasii*	United States
SRR3468048	San_Antonio_1	*Coccidioides posadasii*	United States
SRR3468051	Sonora_1	*Coccidioides posadasii*	Mexico
SRR3468052	Sonora_2	*Coccidioides posadasii*	Mexico
SRR3468033	Tucson_10	*Coccidioides posadasii*	United States
SRR3468034	Tucson_11	*Coccidioides posadasii*	United States
SRR3468035	Tucson_12	*Coccidioides posadasii*	United States
SRR3468036	Tucson_13	*Coccidioides posadasii*	United States
SRR3468037	Tucson_14	*Coccidioides posadasii*	United States
SRR3468039	Tucson_15	*Coccidioides posadasii*	United States
SRR3468040	Tucson_16	*Coccidioides posadasii*	United States
SRR3468041	Tucson_17	*Coccidioides posadasii*	United States
SRR3468042	Tucson_18	*Coccidioides posadasii*	United States
SRR3468043	Tucson_19	*Coccidioides posadasii*	United States
SRR3468044	Tucson_20	*Coccidioides posadasii*	United States
SRR3468045	Tucson_21	*Coccidioides posadasii*	United States
SRR3468046	Tucson_22	*Coccidioides posadasii*	United States
SRR3468047	Tucson_23	*Coccidioides posadasii*	United States
SRR3468073	Tucson_24	*Coccidioides posadasii*	United States
SRR3468032	Tucson_9	*Coccidioides posadasii*	United States
SRR3468031	Tucson_8	*Coccidioides posadasii*	United States
SRR3468030	Tucson_7	*Coccidioides posadasii*	United States
SRR3468029	Tucson_6	*Coccidioides posadasii*	United States
SRR3468028	Tucson_5	*Coccidioides posadasii*	United States
SRR3468026	Tucson_4	*Coccidioides posadasii*	United States
SRR3468025	Tucson_3	*Coccidioides posadasii*	United States
SRR3468024	Tucson_2	*Coccidioides posadasii*	United States
SRR3468023	Tucson_1	*Coccidioides posadasii*	United States
SRR1292228	212	*Coccidioides immitis*	United States
SRR1292225	202	*Coccidioides immitis*	United States
SRR1292226	205	*Coccidioides immitis*	United States
SRR1292227	211	*Coccidioides immitis*	United States
SRR1292224	Washington_1	*Coccidioides immitis*	United States
SRR3468022	B0727_Argentina	*Coccidioides immitis*	Argentina
SRR3468018	Guerrero_1	*Coccidioides immitis*	Mexico
SRR3468019	San_Diego_1	*Coccidioides immitis*	United States
SRR3468021	Coahuila_1	*Coccidioides immitis*	Mexico
SRR3468015	SJV_1	*Coccidioides immitis*	United States
SRR3468016	SJV_2	*Coccidioides immitis*	United States
SRR3468027	SJV_3	*Coccidioides immitis*	United States
SRR3468038	SJV_4	*Coccidioides immitis*	United States
SRR3468049	SJV_5	*Coccidioides immitis*	United States
SRR3468060	SJV_6	*Coccidioides immitis*	United States
SRR3468071	SJV_7	*Coccidioides immitis*	United States
SRR3468079	SJV_8	*Coccidioides immitis*	United States
SRR3468080	SJV_9	*Coccidioides immitis*	United States
SRR3468081	SJV_10	*Coccidioides immitis*	United States
SRR3468017	SJV_11	Coccidioides immitis	United States

To compare the *Coccidioides* mtDNA assembles with other fungi, we retrieved full mitochondrial assemblies from other Onygenales available in the NCBI GenBank database: *Histoplasma capsulatum* H143 (GG692467.1) *Paracoccidioides brasiliensis* Pb18 [AY955840.1, (Cardoso *et al.* 2007)], *Blastomyces dermatitidis* ATCC 18188 (GG753566.1), *Epidermophyton flocossum* ATCC 26072 [AY916130.1, (Tambor *et al.* 2006)], *Trichophyton rubrum* BMU 01672 [FJ385026.1 (Wu *et al.* 2009)], and *Ascosphaera apis* ARSEF 7405 [AZGZ01000045.1 (Shang *et al.* 2016)]. Other close relatives of *Coccidioides* (*e.g.*, *Uncinocarpus*) had fragmented mitogenomes (Whiston *et al.* 2012) and were thus not included in our analyses.

Mitochondrial genes as well as introns of group I and group II, tRNAs, RNaseP RNA (*rnpB*), and the small and large subunit rRNAs (*rns* and *rnl*) for *Coccidioides* and other related Onygenalean fungi were annotated using the MFannot pipeline (https://github.com/BFL-lab/Mfannot). *Coccidioides* annotations were manually inspected and intron boundaries were checked and adjusted by aligning available RNAseq data (Whiston *et al.* 2012) with respective mitochondrial assemblies using Bowtie 2 (Langmead and Salzberg 2012). The assemblies and annotations were deposited in GenBank (MW722165-MW722166) and were visualized with the OGDRAW pipeline (Greiner *et al.* 2019).

### Single nucleotide polymorphism identification

To identify Single Nucleotide Polymorphisms (SNPs) from the 77 *Coccidioides* isolates, we mapped Illumina paired-end reads to the assembled mitochondrial references of either *C. posadasii* strain Tucson2 or *C. immitis* strain WA221 in a species-dependent manner using Burrows-Wheeler Aligner (BWA) v 0.7.7 (Li and Durbin 2009). Indels were realigned to the species reference genomes using GATK RealignerTargetCreator and IndelRealigner tools [GATK toolkit v 3.3-0 (McKenna *et al.* 2010)]. Next, we used the UnifiedGenotyper package. We only included SNPs not located in duplicated loci [as identified by NUCmer (Kurtz *et al.* 2004)], with more than 10X coverage, and with a minor allele frequency of at least 10%. We used the same approach to call SNPs for the nuclear genomes (Teixeira *et al.* 2019a).

### Phylogenetic analysis

We generated maximum likelihood concatenated trees for mtDNA and for nucDNA using methods implemented in IQ-TREE software (Nguyen *et al.* 2015) using -m MFP option [ModelFinder (Kalyaanamoorthy *et al.* 2017)] for model selection. To measure branch confidence, we used 1000 ultrafast bootstraps (Minh *et al.* 2013) and a Shimodaira–Hasegawa-like approximate likelihood ratio test (SH-aLRT) (Anisimova *et al.* 2011). We compared the topology of the two trees using FigTree v1.4.2—http://tree.bio.ed.ac.uk/software/figtree/, and scored the disagreements the two topologies using TOPD/FMTS v 4.6 (Puigbò *et al.* 2007) and the R function *tree.dist* [library *phangorn* (Schliep 2011)]. We calculated the Split Distance (SD) distance score, a metric that ranges between 0 (full concordance) and 1 (complete non-concordance). The SD is the ratio between the observed Robinson-Foulds (RF) symmetric difference and the maximum value of the RF distance (2n-6). Since we had 77 tips, the maximum number of the RF distance is 148. SD ranges from 0 (all partitions present in the two focal trees) to 1 (no shared partitions between the two trees).

### Population genetics

We used fastSTRUCTURE v1.0 (Raj *et al.* 2014) to investigate potential genetic structure at the mitochondrial level within each of the two *Coccidioides* species. We used unlinked SNPs from the final .vcf SNP set using PLINK v1.07 using the –make-bed option. We repeated the same procedure using K (number of populations) values that range from two to eight. We used *chooseK.py* to determine the most likely scenario for the number of genetic clusters.

### Data availability

SNP data for each strain analyzed for nuclear and mitochondrial trees, raw phylogenetic trees files (Figure 2) and unlinked SNPs and allele frequencies used as input for structure analysis (Figure 4) are available at Zenodo (doi:10.5281/zenodo.4592315).

## Results

### *Coccidioides* spp. mitogenome

**Figure 1:**
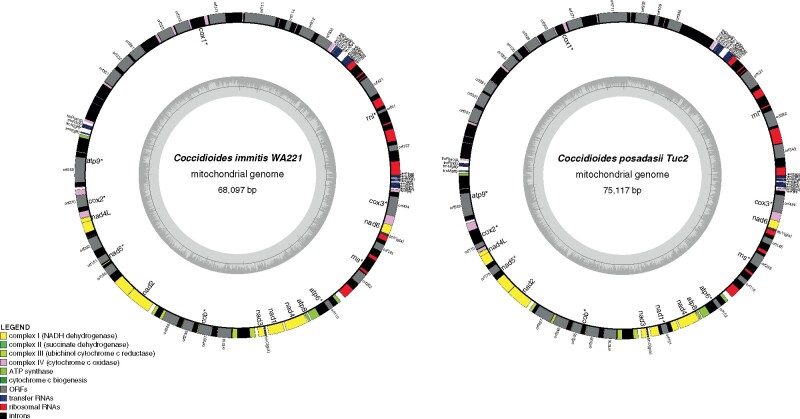
Circular maps of *C. immitis* and *C. posadasii* mitogenomes. The assembled and annotated genome features were converted into GenBank format and loaded into the OGDraw pipeline for physical visualization of the coding and non-coding elements of the mitochondrial genomes.

**Figure 2:**
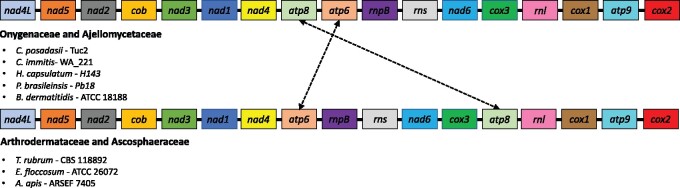
Mitochondrial gene content and synteny among Onygenalean fungi. Genes are color-coded (see legend) and are displayed according to position on the genome. The mitogenomes are highly syntenic but *atp8* gene positioning differs between Onygenaceae/Ajellomycetaceae and Arthrodermataceae/Ascosphaeraceae.

We assembled complete circular mtDNA molecules for each of the two species of *Coccidioides*. The two assemblies differ in size: the mitogenome is 68.6 Kb in *C. immitis* and 75.1 Kb in *C. posadasii* (Figure 1). This observation is consistent with documented variation in mitogenome size among species of the Onygenales (Table 2). The length of the mtDNA of both species of *Coccidioides* are on the larger end of the continuum of sizes of mitogenomes. The mitogenomes of *Coccidioides* harbor 14 protein-coding genes responsible for the formation of ubiquinone oxidoreductase, cytochrome b, cytochrome oxidase, and ATP synthase protein complexes (Figures 1 and 2). The two ribosomal small and large subunit rRNA genes (*rns* and *rnl*), RNase P RNA (*rnpB*) and 26 tRNAs were identified. The gene composition and synteny are conserved between *Coccidioides* (Onygenaceae), *Blastomyces*, *Histoplasma*, and *Paracoccidioides* (all Ajellomycetaceae) (Figure 1; Van Dyke *et al.* 2019). The position of the gene *atp8* differs between the Onygenaceae/Ajellomycetaceae species and other species of the Onygenales, such as dermatophytes (*Trichophyton rubrum* and *Epidermophyton floccosum—*Arthrodermataceae), and the bee-pathogenic fungus *Ascosphaera apis* (Ascosphaeraceae, Figure 2). We observed no gene gain or loss of core mitochondrial genes within Onygenalean fungi (Figure 2).

**Table 2:** Numbers of introns and classes among Onygenalean fungi

	*C. immitis*	*C. posadasii*	*H. capsulatum*	*P. brasiliensis*	*B. dermatitidis*	*E. flocossum*	*T. rubrum*	*A. apis*
Mitochondrial genome size (bp)	68,597	75,194	39,129	71,335	51,071	30,910	26,985	118,650
Intron IB (complete)	15	15	3	5	6	3	0	8
Intron IB (extra insertion)	0	0	1	0	0	0	0	0
Intron IB (5′, partial)	0	1	0	0	0	0	0	1
Intron IB (3′, partial)	1	2	1	0	2	0	0	1
Intron IA	3	3	1	0	1	1	2	1
Intron IA (5′, partial)	0	1	0	0	0	0	0	1
Intron I (derived, A)	1	1	0	0	2	1	0	5
Intron I (derived, B1)	2	2	0	4	0	0	0	11
Intron ID	4	3	1	0	1	1	0	5
Intron IC1	0	0	0	0	0	0	0	2
Intron IC2	4	4	0	1	1	0	0	5
Intron I (derived, B2)	1	1	1	0	1	0	0	1
Intron II (domainV)	4	5	1	3	2	0	0	3
Intron II, derived	1	1	0	0	0	0	0	0
Total	36	39	9	13	16	6	2	44

The larger size of the mitogenome in *Coccidioides* is due to the presence of introns and intron-encoded open reading frames (ORFs) in both *Coccidioides* species, reflected in the increase in the frequency of intron type I and intron type II (Table 2, Figure 1) compared with other Ajellomycetaceae fungi. In fact, *Coccidioides* harbors twice the number of elements found in *B. dermatitidis*. The dermatophyte genera, *Epidermophyton* and *Trichophyton*, have only six and two intron elements respectively, whereas *C. immitis* and *C. posadasii* contain 39 elements, respectively (Table 2). The introns found in the *Coccidioides* mtDNA contain complete or truncated ORFs with high similarity to homing endonucleases of the LAGLIDADG and GIY-YIG families (Table 2). Both species contain 15 complete copies of Intron IB (Table 2). *Ascopharaceae apis* also has a large mitogenome (118.65 Kb) and a high number of intron-type I, specifically the intron I—derived, B1 element (Table 2). The frequency and distribution of intron-types I and II in the genes *nad5*, *cob*, and *cox1* differ between *C. immitis* and *C. posadasii* (Figure 1). The difference in genome size between *Coccidioides* species stems from the increased number of copies of intron I and intron IB (Table 2) and repetitive DNA between ORF 330 and tRNA-H (Figure 1) in *C. posadasii*.

### mtDNA and nucDNA phylogenetic trees

**Figure 3:**
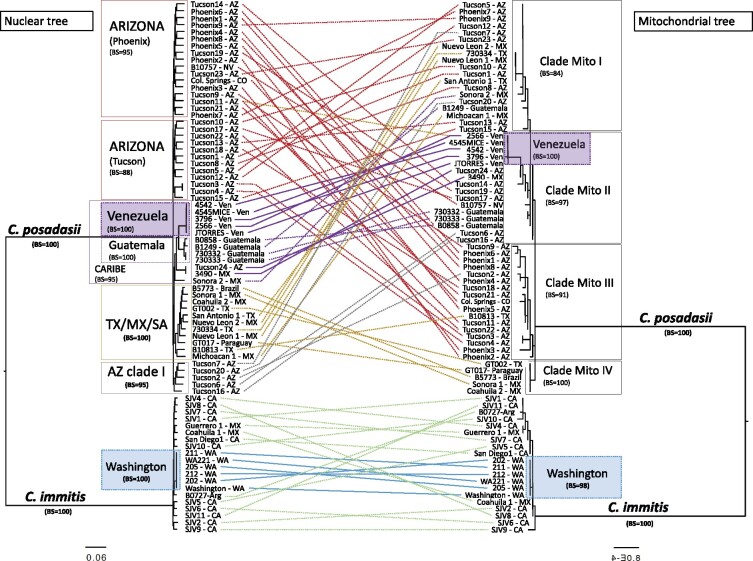
Tree topology comparisons of *Coccidioides* nucDNA (left panel) and mtDNA (right panel) phylogenomic trees. Phylogenetic tree branches are proportional to the nucleotide divergence (see scale) and the main clades are highlighted. Bootstrap support was calculated, and branch support was added to the corresponding clade. The terminal taxa are color-coded according to their placement on the nucDNA tree and taxa are connected between mtDNA and nucDNA phylogenomic trees to visualize concordance (solid lines) *vs* discordance (dotted lines).

To determine whether the nuclear and the mitochondrial genomes show the same evolutionary trajectories, we compared the mitochondrial phylogeny with the whole genome species phylogeny. To score the differences in partitions produced between mtDNA and nucDNA trees, we used a normalized RF symmetric distance (Split distance, SD). If two trees are completely congruent the split distance score is 0, whereas with complete tree disagreement the score is 1. In the case of the nucDNA *vs* mtDNA tree comparison, the split distance equals 0.92 [138/150] thus indicating that the topologies are largely inconsistent with each other. Both topologies support species divergence between *C. immitis* and *C. posadasii* (*i.e.*, no isolates being assigned to a different species; see Figure 3) but little to no concordance within species, especially within *C. posadasii*. Nonetheless, the lengths of the branches for each of the two species are much shorter than expected. This is unexpected because the mutation rate of mtDNA is larger than the mutation rate of nucDNA. In other fungi, mutation rates are orders of magnitude larger than in nuclear genes (Chou and Leu 2010). We assumed a nuclear mutation rate of 1 × 10^−11^ per site per generation (the order of magnitude in *Aspergillus flavus*, *A. fumigatus*, and *A. nidulans*) and a mitochondrial mutation rate of 0.76 to 1.6 × 10^−7^ per site per generation, which is a rate that seems to be consistent across most eukaryotes (Konrad *et al.* 2017). These rates suggest that the divergence of the nuclear genomes occurred approximately 1 × 10^9^ generations, while the divergence of the mitochondrial genomes occurred in approximately 1 × 10^4^ generations. This result suggests ancient exchange of mitochondrial genomes. The species-level concordance indicates that the mtDNA exchange event is old enough that each species has reaccumulated sufficient substitutions to differentiate the two species.

**Figure 4:**
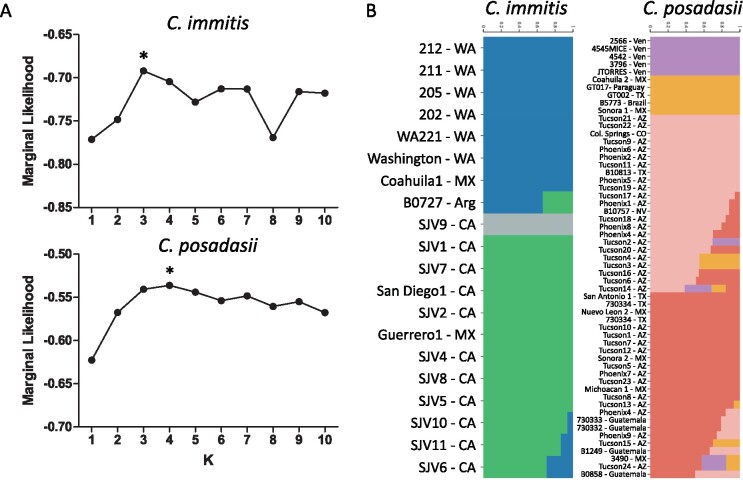
Population structure within *C. immitis* and *C. posadasii* based on mitochondrial-derived alleles. (A) Marginal likelihood plots for both *C. immitis* and *C. posadasii* species and the best K (or number of populations) selected within each species is marked with asterisks and was *K* = 2 and *K* = 4, respectively. (B) Proportion of admixture of each *C. immitis* or *C. posadasii*. Heterozygous or non-admixed strains are represented by solid bar while admixed individuals are displayed as multicolored bars. The proportion of each homozygous population can be observed by the scale on the top of each plot.

Next, we studied the arrangement of variation within each of the two species. Even though the species-level split was consistent between nucDNA and mtDNA, the topologies were largely inconsistent with each other for within-species comparisons. For *C. posadasii*, previous phylogenetic analyses of nuclear markers have shown that at least three distinct populations exist: isolates from Arizona (clinical and environmental), isolates from Texas, Brazil, Argentina, and Mexico, and a third set of isolates from the Caribbean region (Engelthaler *et al.* 2016; Teixeira *et al.* 2019a). Using mtDNA markers, we identified four clusters that do not follow this strong biogeographic pattern. For example, the isolates B10813, Tucson2 and Tucson20 have conflicting phylogenetic distributions, as previously these were placed within the *Texas/Mexico/SouthAmerica* or *AZ clade I* using nuclear markers (Figure 3). *Mito1* is composed of 19 isolates from four populations as defined by nuclear markers (*Arizona*, *Texas/Mexico/SouthAmerica*, *Caribbean* and *AZ clade I*); *Mito2* is composed of 16 isolates composed mostly of isolates from the Caribbean region—including a monophyletic group from Venezuela, and four isolates from *Arizona* and two from *AZ clade I*; *Mito3* is composed of 17 isolates, mostly from Arizona (*Arizona* and *AZ clade I*) but with one isolate from Texas (*Texas/Mexico/SouthAmerica* population), and *Mito4* is composed of fiveisolates, all from the *Texas/Mexico/SouthAmerica* population. These four clusters were also confirmed by FastStructure (Figure 4). For *C. immitis* both nucDNA and mtDNA phylogenies revealed a clade composed of isolates from Washington, which is genetically distinct from the rest of *C. immitis* (Figure 3). No other consistent pattern of clustering was observed for the remaining *C. immitis* individuals comparing the two phylogenies (Figure 3).

Using marginal likelihoods to assess the number of populations, three groups were observed within *C. immitis*, and four groups were supported in *C. posadasii* (Figure 4A). For *C. immitis* the three groups are (1) a group containing the Washington state, Argentina, and Coahuila1 isolates (Figure 4B, blue); (2) a group with a single strain, SJV9 (Figure 4B, grey); and (3) a third group composed of isolates from San Joaquin Valley, San Diego and Mexico (Figure 4B, green). Within *C. posadasii*, the four groups supported are: (1) a group containing all the Venezuela isolates (Figure 4B, purple); (2) a group formed with strains from *Texas/Mexico/South America* nucDNA clade, and corresponding to the clade *Mito IV* in the phylogenetic tree (Figure 4B, yellow); (3) a group formed by strains from Arizona and mostly belonging to the *Tucson* nucDNA clade (Figure 4B, pink); and (4) a group composed of the remaining isolates from Guatemala, Arizona, Mexico, and Texas (Figure 4B, salmon). This last group does not correspond to the population structure defined for nucDNA genes. We observed some admixed genotypes within *C. immitis*, and several within the *C. posadasii* species suggesting that gene flow in the mitogenome might occur within these groups (Figure 4B, isolates with mixed color bars).

## Discussion

Mitochondrial genomes are responsible for key aspects of cellular metabolism. In this article, we report the assembled mitochondrial genomes of two species of the human pathogen *Coccidioides* and study the extent of genetic variation in the mtDNA of these two species. Our results have two implications. First, the assembly of the full circular genomes allows us to study conservation of gene order, content, and size among the Onygenales. Second, the study of polymorphism within and among species allows us to determine whether mtDNA markers are suitable for species and population identification. We discuss each of these implications as follows.

### Gene order conservation

Gene content in mitochondria is remarkably conserved across the tree of life. However, fungal genomes show differences in the order of gene arrangement. Our results suggest that there is complete synteny in the mtDNA genome of the species from the Onygenaceae family in spite of over 100 million years of divergence. Howevr, this synteny is not conserved for the entire Onygenales order. Members of the family Arthrodermataceae and Ascosphaeraceae show similar gene order, but reveal a different order compared with Onygenaceae and Ajellomycetaceae (Figure 2). The *atp6*-*atp8* pair is a highly conserved syntenic unit across the Eurotiomycetes (Aguileta *et al.* 2014), but the pair is disrupted by genomic rearrangements found within Arthrodermataceae and Ascosphaeraceae. This level of conservation over 150 million years [the crown age for the Onygenaceae family (Sharpton *et al.* 2009; Rouxel *et al.* 2011)] is comparable with the conservation of mtDNA order in a different group of ascomycetes, the species from the order Hypocreales [crown age: 179 MYA, (Sung *et al.* 2008; Rouxel *et al.* 2011)], but dissimilar to the level of turnover that occurs among other fungi (Aguileta *et al.* 2014). This apparent difference in the rate of evolution of gene order in mtDNA across taxa could be real, or be caused by unequal taxonomic sampling. The mechanisms for fungal mitochondrial gene order evolution remain largely unknown, but have been attributed primarily to mitochondrial non-homologous recombination (Beaudet *et al.* 2013). Gene order turnover and stasis could then be attributed to differences in the rate of mtDNA recombination. While this is a feasible explanation, only a systematic study with balanced sampling across fungi will resolve whether there are differences in the rate of evolution in gene order in fungal mitochondrial genomes.

### Mitochondrial polymorphism

Molecular markers from mitochondrial genomes are extensively used as molecular markers in speciation studies (Dupuis *et al.* 2012). Before this study, the mitochondrial genome and the phylogenetic relationships of mitochondrial haplotypes in *Coccidioides* remained unstudied. Our results suggest that mtDNA can be used to distinguish between *Coccidioides* species, and that species-level diagnostic efforts using mtDNA variation are well-founded (Hamm *et al.* 2019). Nonetheless, mtDNA markers might lead to incorrect assignments at the intraspecies population level due to extensive genealogical incongruence within species, particularly within *C. posadasii*. Conflicting phylogenetic histories in mtDNA and nucDNA genealogies have been observed in other pathogenic fungi. For example, *Paracoccidioides brasiliensis* and *P. restrepiensis* appear to be polyphyletic using mtDNA markers, and the tree topologies differ from those obtained from nucDNA markers (Turissini *et al.* 2017). The incongruence between mtDNA and nucDNA in *Paracoccidioides* species seems to be the result of interspecific hybridization (Turissini *et al.* 2017). The remnants of such admixture events are only observed in the mtDNA as most of the nucDNA shows no evidence of gene exchange (Mavengere *et al*. 2020). Genealogical discordance between mitochondrial and nuclear gene genealogies also occurs in the *Cryptococcus gattii* species complex and seems to be caused by interspecies introgression (Bovers *et al.* 2009). Blocks of mtDNA with mixed ancestry have been observed in *Candida* (Anderson *et al*. 2001), *Verticillum* (Depotter *et al.* 2018), and *Phellinus* (Lee *et al.* 2019). The exchange of mtDNA across fungal species remains largely understudied, but represents an important source of genetic variation and evolutionary history. Additional admixture analyses within *Coccidioides* species are required to further evaluate the ancestral traces between different genotypes. Certainly, there are nuclear mitochondrial (NUMTs) genes found in the nuclear genome, and mapping analysis will be inconclusive unless the *bona fide* NUMT sequences have significantly diverged, or if long reads clearly identify the inserts in nucDNA to correct short read error with confidence. Although our SNP analysis method removed low coverage SNPs and rare alleles, and coverage of the mitogenome was very high, it remains a formal possibility that some of the SNPs are due to NUMTs. Thus, future mtDNA and nucDNA analysis in both species of *Coccidioides* should consider long read sequences to more fully explore this issue.

Pioneering studies of fungal mitochondrial polymorphisms suggest a lower rate of nucleotide substitutions in the mitochondrial genomes compared with mammals (Clark-Walker 1991). Population demographics and ecological factors can modulate the lower amount of mtDNA mutations in fungal species (Basse 2010). Despite the high levels of divergence between nuclear-mitochondrial trees within *C. posadasii*, we have observed low mitochondrial diversity within some genetic groups; there is a little to no mitochondrial genetic variation between the individuals Tucson5, Phoenix7, Phoenix9, Tucson12, Tucson7, and Tucson23 within the *C. posadasii* Mito1 clade compared with the genome tree. In contrast, the individuals from the Venezuela group also show a lower intraclade variation but are concordant nuclear-mitochondrial tree topologies. This pattern is also observed in other fungal species. Within the two dominant *C. gatti* lineages VGI and VGII, the mitochondrial sequence divergence within groups was about three times lower compared with nuclear genes (Xu *et al*. 2009). This observation suggests that selective sweeps in fungal mitogenomes are potentially due to uniparental inheritance of mitochondrial DNA due to the dominance of a few mitotypes in natural fungal populations.

Mitochondrial genomes may be associated with hybrid incompatibilities. Among hybrids between different species [*e.g.*, *Saccharomyces* (Lee *et al.* 2008; Chou *et al.* 2010; Chou and Leu 2010); *Drosophila* (Montooth *et al.* 2010; Clancy *et al.* 2011)], and even within species [*e.g.*, *Caenorhabditis* (Chang *et al.* 2016), *Tigriopus copepods* (Barreto *et al.* 2018; Lima *et al.* 2019)], epistatic interactions between the nucDNA and the mtDNA from progenitors can lead to hybrid breakdown, which in turn might be of importance for the persistence of species in the face of secondary contact and gene flow. The interaction between mtDNA and nucDNA plays a role on the fitness of fungal interspecific hybrids (Olson and Stenlid 2001; Giordano *et al.* 2018). Notably, alleles involved in reproductive isolation are more likely to show gene genealogies that deviate from the species tree, which makes mtDNA gene genealogies an unreliable proxy of evolutionary history in cases in which there are mito-nuclear incompatibilities. Understanding whether or not these interactions are pervasive across fungi will require the production of interspecific hybrids and the systematic study of their fitness, a task currently not feasible for *Coccidioides* (Burt *et al.* 1996; Chou and Leu 2010).

### Future directions

The endozoan lifestyles of *Coccidioides* spp. (Taylor and Barker 2019) suggest that mitochondrial genes involved in thermal adaptation and oxidative stress could be under strong directional selection. Mitochondria are the primary site of ATP production, and are possibly associated with organismal thermal tolerance; however, general principles governing these patterns remain undefined (Chung and Schulte 2020). For example, the size of pathogenic nematode mitogenomes from different thermal habitats is significantly smaller in endothermic host species comparing with ectothermic hosts suggesting that host adaptation might be a significant impact on mitochondrial evolution (Lagisz *et al.* 2013). *Coccidioides posadasii* is more heat tolerant than *C. immitis* (Mead *et al*. 2020). In *Saccharomyces*, polymorphisms in the *cox1* gene of mtDNA are associated with adaptation to variable temperatures. The yeast mitochondrial genome might be a hotspot in the evolution of thermal adaptation in *Saccharomyces* species (Baker *et al.* 2019; Li *et al.* 2019). In *Cryptococcus*, highly virulent strains show an increased ability to replicate within macrophages, which is in turn associated with an unusual mitochondrial morphology and upregulation of genes encoded by the mitochondrial genome and associated with mitochondrial activities (Ma *et al.* 2009; Ma and May 2010). Whether mtDNA variation is associated with thermal fitness or virulence in *Coccidioides* remains to be tested. Our genome assembly and measurements of variation also open the door to study the evolutionary drivers of mitochondrial variation in *Coccidioides*. Genealogical discordance is higher with *C. posadasii* than within *C. immitis* which might suggest differences in the rate of mtDNA recombination between species. These potential differences in recombination might indicate that the genomes of the two *Coccidioides* species might be under different selection regimes, which in turn might also differ from other fungi (Sandor *et al.* 2018).

## Conclusions

The assembly and measurement of polymorphism in the mitogenome of *Coccidioides* will facilitate deeper investigations into the impact of mitochondrial evolution in *Coccidioides’* niche adaptation, with particular emphasis on mammalian host co-evolution and oxidative stress responses. The results shown here will also aid in the study of the evolutionary drivers of mitogenome evolution in fungi.
